# A multi-dimensional nomogram to predict non-sentinel lymph node metastases in T1–2HR+ breast cancer

**DOI:** 10.3389/fendo.2023.1121394

**Published:** 2023-07-05

**Authors:** Ke Xiang, Jialin Chen, Yu Min, Hang Chen, Jiaxin Yang, Daixing Hu, Yuling Han, Guobing Yin, Yang Feng

**Affiliations:** ^1^ Department of Breast and Thyroid Surgery, The Second Affiliated Hospital of Chongqing Medical University, Chongqing, China; ^2^ The Second Affiliated Hospital of Chongqing Medical University, Chongqing, China

**Keywords:** breast cancer, nomogram, axillary non-SLN metastases, pathological features, survival analysis

## Abstract

**Background:**

Axillary lymph node dissection (ALND) could be omitted for T1-2 breast cancer patients with 1-2 positive sentinel lymph node (SLN) after breast-conserving surgery when radiation is planned. However, whether ALND could be replaced by radiation in patients with 1-3 positive SLNs when no more non-SLN metastasis were observed after mastectomy are still controversial. The aim of our study was to develop and validate a nomogram for predicting the possibility of non-SLN metastasis in T1–2 and hormone receptor (HR) positive breast cancer patients with 1-3 positive SLNs after mastectomy.

**Methods:**

We retrospectively reviewed and analyzed the data including the basic information, preoperative sonographic characteristics, and pathological features in breast cancer patients with 1-3 positive SLNs in our medical center between Jan 2016 and Dec 2021. The Chi-square, Fisher’s exact test, and t test were used for comparison of categorical and qualitative variables among patients with or without non-SLN metastasis. Univariate and multivariate logistic regression were used to determine the risk factors for non-SLN metastasis. These predictors were used to build the nomogram. The C-index and area under the receiver operating characteristic curve (AUC) was calculated to assess the accuracy of the model.

**Results:**

A total of 49 in 107 (45.8%) patients were identified with non-SLN metastasis. In multivariate analysis, four variables including younger age, lower estrogen receptor (ER) expression, higher histological score, and cortex thickening of the lymph nodes were determined to be significantly associated with non-SLN metastasis. An individualized nomogram was consequently established with a favorable C-index of 0.822 and verified *via* two internal validation cohorts.

**Conclusions:**

The current study developed a nomogram predicting non-SLN metastasis for T1–2 and HR+ breast cancer with 1–3 positive SLNs after mastectomy and found that patients in the high-risk group exhibited worse relapse-free survival. The novel nomogram may further help surgeons to determine whether ALND could be omitted when 1-3 positive SLNs were observed in T1–2 and HR+ breast cancer patients.

## Introduction

For traditional radical mastectomy, as the standard treatment, axillary lymph node dissection (ALND) is an indispensable part of the operation in patients with axillary negative or positive (1–5). The impact of ALND is to assist clinicians in planning their treatment and to provide more information on prognosis. However, ALND may result in pain, numbness, paresthesia, arm/shoulder mobility restriction, and lymphedema (6–8). Breast lymph is drained *via* two main pathways: axillary nodes and internal nodes (9, 10). Nowadays, as the first station lymphatic node receiving lymphatic drainage from primary breast tumors, the sentinel lymph node biopsy (SLNB) has become a standard tool for evaluating axillary lymph node status in early breast cancer. Breast cancer patients who have negative sentinel lymph node (SLN) could avoid ALND (2–5, 11–17). However, when limited positive SLN were observed, the best treatment for the armpit depends. Based on ACSOG Z0011, AMAROS and OTOASOR study, axillary recurrence (RR) and overall survival (OS) did not differ in patients with low tumor burden and pathologically confirmed 1-2 positive SLN with or without ALND when breast-conserving surgery and radiation were conducted (1, 2, 4, 12, 18–23). The OTOASOR trial compares completion of axillary lymph node dissection (ALND) to regional nodal irradiation (RNI) in patients with sentinel lymph node metastasis (pN1 SLN) in stage I-II breast cancer. The long term follow-up results of this prospective-randomized trial suggest that RNI without ALND does not increase the risk of axillary failure in selected patients with early-stage invasive breast cancer (cT ≤ 3 cm, cN0) and pN1(SLN). Patients with T1-2 primary breast cancer and no palpable lymphadenopathy were enrolled in AMAROS trial. Both trials included post-mastectomy patients, but no overall or progression-free survival data were reported. By this token, for early breast cancer patients with 1-3 positive SLNs after mastectomy, the effect of ALND is still controversial and may be closely related to the number of lymph node metastases.

Previously, some single-center or public databases found compared with HR+ breast cancer subtypes, HER2 over expression and basal-like subtypes have a worse prognosis (24–27). In clinical practice, we may found a considerable proportion of patients with 1-3 positive SLNs, while no more non-SLN metastasis was observed when ALND was conducted. Therefore, it is necessary to identify patients at high risk of non-SLN metastasis that might benefit from ALND, and those low risk patients could avoid overtreatment and procedural complications. Up to now, there have been few studies that attempt to develop easily recognizable risk stratification or prognostic model for T1–2 and HR+ breast cancer patients with 1-3 positive SLNs after mastectomy, the purpose of this study is to determine risk factors for non-SLN metastasis in T1–2 and HR+ breast cancer patients with 1-3 positive SLNs after mastectomy and to develop a nomogram prediction model.

## Methods

### Study design and patient cohort

**Figure 1:**
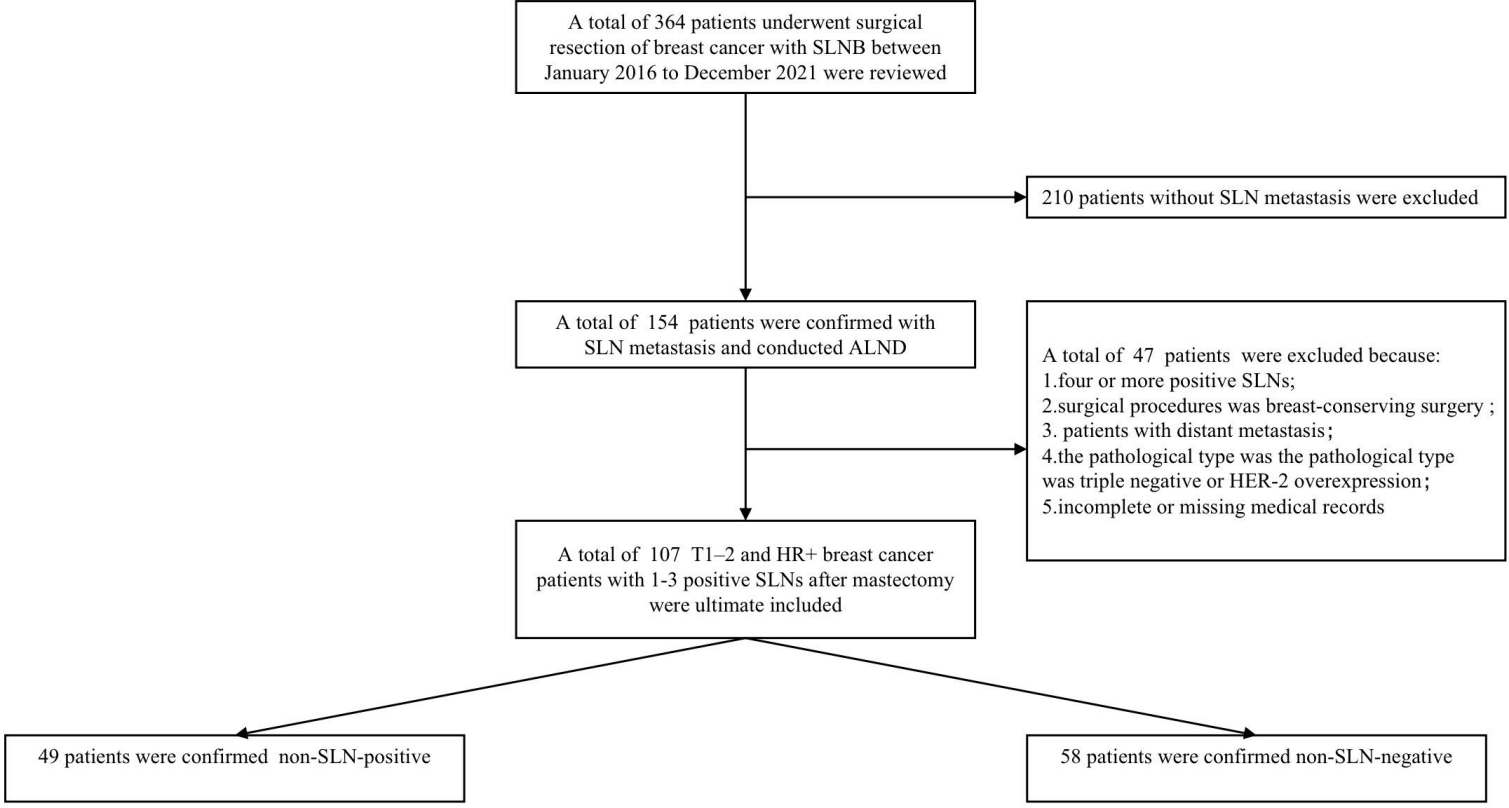
The patient selection process for the present study. Non-SLN-negative defined as positive axillary lymph nodes in patients with 1-3 positive SLNs. Non-SLN-positive defined as the non-sentinel metastasis in patients with 1-3 positive SLNs.

The data of T1–2 and HR+ breast cancer patients with 1-3 positive SLNs between Jan 2016 and Dec 2021 were derived from the Department of Breast and Thyroid Surgery, the Second Affiliated Hospital of Chongqing Medical University. Ethical approval was approved by the local Ethics Committee of the Chongqing Medical University. The specific including and excluding criteria were summarized in **Figure 1**. Criteria for inclusion were as follows: 1) invasive breast cancer confirmed by preoperative puncture histology, 2) the preoperative clinical diagnosis was T1-2, N0-N1 according to the 8th American Joint Committee on Cancer (AJCC), 3) HR+ was confirmed by immunohistochemistry before operation, 4) intraoperative frozen section confirmed 1-3 positive SLNs, 5) the surgical procedure was mastectomy with ALND, 6) all patients received radiotherapy after operation. Patients don’t met this criteria were excluded.

### Data collection

Data on clinicopathological and treatment procedures were collected from eligible patients’ medical records, including cT, cN, tumor side (left and right), age, BMI, neoadjuvant chemotherapy (NACT) (yes and no), number of positive SLN (1–3), histological score (HS), estrogen receptor (ER), progesterone receptor (PR), human epidermal growth factor receptor 2 (HER-2) and Ki-67. HER-2 immunohistochemical staining was scored from 0 to 3+, 0 or 1+ were considered negative, and HER-2 protein 3+ or HER-2 gene amplification was defined as HER-2 positive. Characteristics of the color doppler ultrasound included tumor location (upper outer quadrant, upper inner quadrant, lower inner quadrant, lower outer quadrant, and central), multifocality, distance between tumor and nipple, long axis, short axis, blood flow (yes and no), calcification(yes and no), cortex(thickening and normal), lymphatic hilum (disappear and normal) of the lymph nodes.

### Statistical analysis

The correlation between different clinicopathological variables in non-SLN positive and non-SLN negative group were analyzed. Tests of Fisher exact or Pearson chi-square were used to analyze categorical variables. Quantitative variables were analyzed by Student’s two-tailed t-test. The risk factors were analyzed using both univariate and multivariate logistic regressions. Factors with a p-value < 0.2 on univariate analysis were included in the multivariate model. A nomogram for predicting non-SLN metastasis in T1–2 and HR+ breast cancer patients with 1-3 positive SLNs after mastectomy based on the results of the multivariate logistic regression analysis was developed and evaluated by two cohorts, and calibration curves as well as decision curve analysis (DCA) curve were plotted to assess the performance and clinical application value of the nomogram. All analyses were performed using the SPSS26 (SPSS/IBM, Chicago, IL, USA) and R 4.2.1 software.

## Results

### Clinical characteristics

Generally, 107 patients were ultimately enrolled in this study. Patient demographics and clinical characteristics are shown in **Table 1**. The patients with non-SLN positive involvement had younger age, a higher HS, more enlarged lymph nodes, and lower levels of ER expression.

**Table 1:** Baseline characteristics of non-SLN positive and non-SLN negative patients.

Variables	Subgroup	No. of patients		
		Non-SLN positive (n=49, 45.8%)	Non-SLN negative (n=58, 54.2%)	P
**Side**	Left	24 (22.4%)	32 (29.9%)	0.564
	Right	25 (23.4%)	26 (24.3%)	
**Age (Mean±SB, years)**		48.7 (±11.7)	53.2 (±10.3)	**0.034**
**BMI (Mean± SB， kg/m^2^)**		23.1 (±2.6)	23.4 (±2.4)	0.515
**NACT**	Yes	25 (23.4%)	25 (23.4%)	0.442
	No	24 (22.4%)	33 (30.8%)	
**Number of positive SLN**	1	21 (19.6%)	36 (33.6%)	0.120
	2	17 (15.9%)	15 (14.0%)	
	3	11 (10.3%)	7 (6.5%)	
**Tumor location**	Upper outer quadrant	19 (17.8%)	33 (30.8%)	0.168
	Upper inner quadrant	10 (9.3%)	9 (8.4%)	
	Lower inner quadrant	3 (2.8%)	6 (5.6%)	
	Lower outer quadrant	6 (5.6%)	5 (4.7%)	
	Central	11 (10.3%)	5 (4.7%)	
**Multifocality**	1	45 (42.1%)	55 (51.4%)	0.800
	2	3 (2.8%)	2 (1.9%)	
	3	1 (0.9%)	1 (0.9%)	
**Distance (Mean±SB, mm)**		18.8 (±13.5)	18.8 (11.9)	0.995
**Long axis of LN (Mean±SB,mm)**		13.3 (±11.8)	6 (±7.7)	**<0.001**
**Short axis of LN (Mean±SB,mm)**		7.5 (±6.7)	3.8 (±4.9)	**0.001**
**Blood flow**	No	29 (27.1%)	12 (11.2%)	**0.034**
	Yes	20 (18.7%)	46 (43.0%)	
**Calcification**	No	47 (43.9%)	1 (0.9%)	0.595
	Yes	2 (1.9%)	57 (53.3%)	
**Cortex**	Normal	20 (18.7%)	11 (10.3%)	**<0.001**
	Thickening	29 (27.1%)	47 (43.9%)	
**Lymphatic hilum**	Normal	26 (24.3%)	44 (41.1%)	**0.016**
	Disappear	23 (21.5%)	14 (13.1%)	
**Histological score**	5	0	2 (1.9%)	**0.009**
	6	20 (18.7%)	38 (35.5%)	
	7	26 (24.3%)	14 (13.1%)	
	8	3 (2.8%)	4 (3.7%)	
**ER (Mean±SB)**		66 (±27)	76 (±22)	**0.046**
**PR (Mean±SB)**		47 (±31)	46 (±36)	0.864
**HER-2**	Positive	12 (11.2%)	9 (8.4%)	0.329
	Negative	37 (34.6%)	49 (45.8%)	
**KI-67 (Mean±SB)**		36 (±21)	35 (±26)	0.836
**cT**	1	17 (15.9%)	28 (26.2%)	0.174
	2	32 (29.9%)	30 (28.0%)	
**cN**	0	14 (13.1%)	35 (32.7%)	**0.002**
	1	35 (32.7%)	23 (21.5%)	

BMI, body mass index; NACT, neoadjuvant chemotherapy; ER, estrogen receptor; PR, progesterone receptor; HER-2, human epidermal growth factor receptor 2; cT, clinical T stage; cN, clinical N stage.

Bold values representing statistical significance.

### Univariate and multivariate logistic regression analysis

A total of 21 selected indicators including cT, cN, side, age, BMI, NACT, number of positive SLN, tumor location, multifocality, HS, ER, PR, HER2, ki-67, distance between tumor and nipple, long axis, short axis, blood flow, calcification, cortex, lymphatic hilum of the lymph nodes. The specific value of each variable is summarized in **Table 2**. During the univariate logistic regression analysis, cN (p = 0.001), age (p < 0.037), number of positive SLN (p = 0.125), HS (p = 0.017), ER (p = 0.05), long axis of lymph nodes (p = 0.01), short axis of lymph nodes (p = 0.003), blood flow (p = 0.026), cortex (p < 0.001) and lymphatic hilum (p = 0.015) were significantly associated with non-SLN positive. In multivariate logistic regression analyses, three variables including age (HR = 0.929, 95% CI: 0.882–0.979, p = 0.006), ER (HR = 0.974, 95%CI: 0.953–0.995, p = 0.015), and cortex (HR =10.545,95%CI: 1.526–72.842, p = 0.017) were the independent risk factors, HS (HR =2.246, 95%CI: 0.981–5.14, p = 0.056) reached marginal significance.

**Table 2:** Univariate and multivariate logistic regression analysis of 107 patients with non-SLN positive lymph nodes in T1-2 and HR+ breast cancer with 1-3 positive SLNs after mastectomy.

Variables	Subgroup	Univariable		Multivariable	
		Hazard ratio	P	Hazard ratio	P
**Side**	Left	1	0.523		
	Right	1.282 (0.598, 2.748)			
**Age**		0.962 (0.028, 0.998)	**0.037**	0.929 (0.882, 0.979)	**0.006**
**BMI**		0.949 (0.812, 1.109)	0.512		
**NACT**	Yes	1.375 (0.64, 2.952)	0.414		
	No	1			
**Number of positive SLNB**	1	1	0.125		
	2	1.943 (0.807, 4.677)			
	3	2.694 (0.906, 8.011)			
**Tumor location**	Upper outer quadrant	1	0.187		
	Upper inner quadrant	1.93 (0.667, 5.586)			
	Lower inner quadrant	0.868 (0.194, 3.878)			
	Lower outer quadrant	2.084 (0.56, 7.757)			
	Central	3.821 (1.153, 12.66)			
**Multifocality**	1	1	0.804		
	2	1.833 (0.293, 11.452)			
	3	1.222 (0.74, 20.092)			
**Distance**		1 (0.97, 1.031)	0.995		
**Long axis of LN**		1.08 (1.034, 1.129)	**0.010**		
**Short axis of LN**		1.116 (1.039, 1.199)	**0.003**		
**Blood flow**	No	1	**0.026**		
	Yes	2.644 (1.126, 6.206)			
**Calcification**	No	1	0.475		
	Yes	2.426 (0.213, 27.588)			
**Cortex**	Normal	1	**<0.001**	10.545 (1.526, 72.842)	**0.017**
	Thickening	6.195 (2.598, 14.776)			
**Lymphatic hilum**	Normal	1	**0.015**		
	Disappear	2.78 (1.221, 6.328)			
**Histological score**		2.158 (1.15, 4.05)	**0.017**	2.246 (0.981, 5.144)	**0.056**
**ER**		0.984 (0.969, 1)	**0.05**	0.974 (0.953, 0.995)	**0.015**
**PR**		1.001 (0.99, 1.012)	0.862		
**HER-2**	Positive	1.766 (0.673, 4.63)	0.248		
	Negative	1			
**KI-67**		1.002 (0.986, 1.018)	0.834		
**cT**	1	1	0.158		
	2	1.757 (0.804, 3.84)			
**cN**	0	1	**0.001**		
	1	3.804 (1.687, 8.577)			

Bold values representing statistical significance.

### Nomogram construction and validation

**Figure 2:**
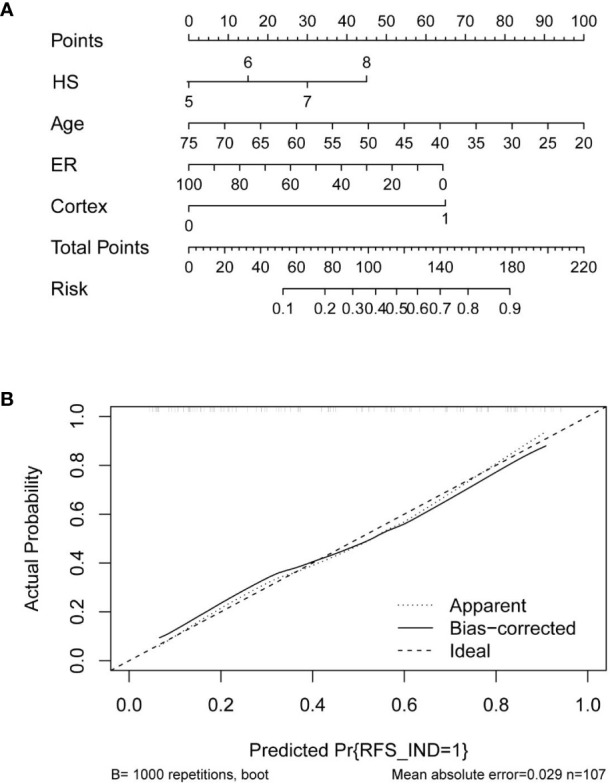
**(A)** Clinical factor-based nomogram used for preoperatively predicting the possibility of four or more positive nodes in T1-2 and HR+ breast cancer patients with 1-3 positive SLNs. **(B)** The calibration curves in the training cohort.

**Figure 3:**
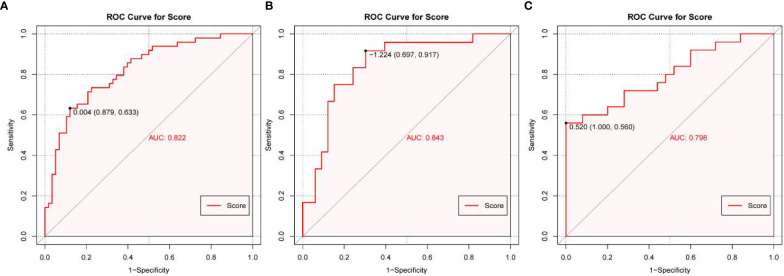
The receiver operating characteristics (ROC) curve and area under the ROC curve (AUC). **(A)** The ROC in the training cohort, **(B)** The ROC in the first validating cohort; **(C)** The ROC in the second validating cohort.

**Figure 4:**
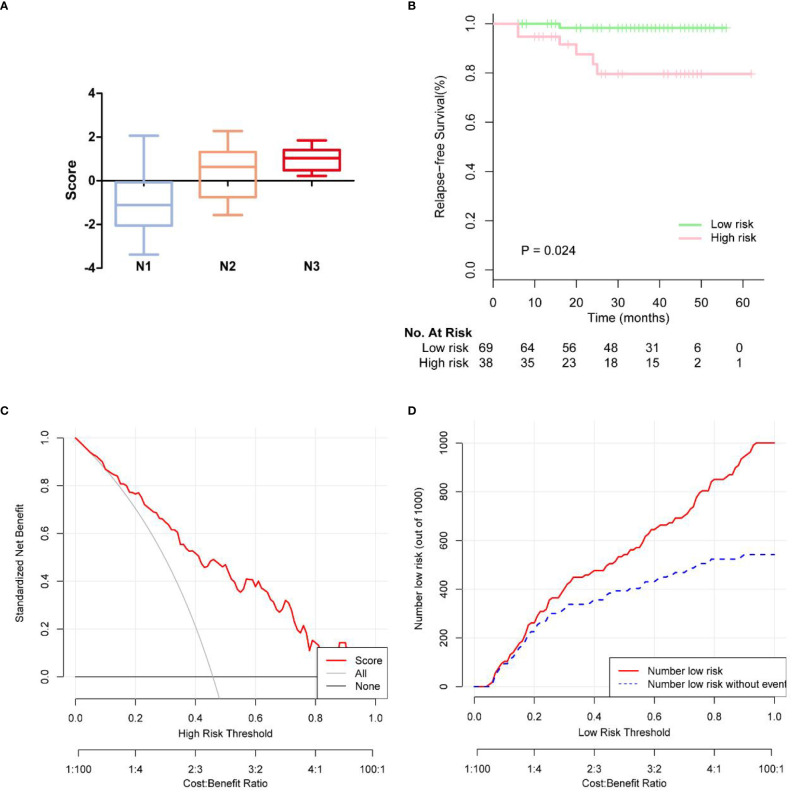
**(A)** Box plots showing patterns of correlation between pathologic N stages and score of rist. **(B)** KM survival curves of disease-free survival on the follow-up low-risk and high risk breast cancer patients. **(C)** The calibration curves for evaluating the accuracy of the nomogram and determination of decision points via Decision Curve Analysis (DCA). **(D)** Clinical impact curve for the risk model.

Based on the multivariate logistic regression analysis results, four variables including age, ER, cortex thickening of the lymph nodes, and HS were used to construct an intuitive nomogram for predicting non-SLN metastasis in T1–2 and HR+ breast cancer patients with 1-3 positive SLNs after mastectomy (**Figure 2A**). The calibration curve (**Figure 2B**) suggested the mean absolute error of training model was 0.017.An optimistic C-index of 0.822 was achieved, which was in accordance with the AUC (**Figure 3A**). The model was further validated by two independent cohorts stratified by whether NACT was conducted, which achieved a AUC value of 0.843 (**Figure 3B**) and 0.798 (**Figure 3C**), respectively. In addition, the decision curve analyses (DCA) were performed to evaluate the utility of the model in detecting non-SLN metastasis in patients with 1-3 positive SLNs. As shown in **Figure 4C**, The DCA indicated that nomogram could add more net benefits than a treat-none or treat-all strategy with the threshold probability range from 0 to 1.0.In addition, we planned to plot clinical impact curve (CIC) to validate the model (**Figure 4D**).

### Survival analysis

In 107 patients, the median follow-up time was 37.7 months. The endpoints were locoregional recurrence (LRR) or distant metastasis (DM). LRR occurred in 2 of 107 (1.9%) patients in ipsilateral chest wall. Distant metastases (DM) occurred in 5 of the 107 (4.7%) patients. In **Figure 4A**, we observed more advanced N stage with increasing score. We further generated Kaplan-Meier (KM) survival curves to compare relapse-free survival (RFS) between low risk and high risk group in **Figure 4B**. Low risk group had 2-year RFS of 98.3%, whereas patients with high risk group had 2-year RFS of 79.5% (p = 0.024).

## Discussion

At present, the overall trend of axillary treatment for patients with early breast cancer is to pursue accuracy and safety on the basis of ensuring effective disease control, and to improve the long-term quality of life of patients through axillary surgery subtraction. SLNB have become the preferred method of staging in patients with clinically negative axillary lymph nodes (2, 11, 12). Fu et al. compared 214 patients with primary invasive breast cancer, patients with pathologically confirmed N1 stage undergoing mastectomy. The results showed that there was no significant difference in OS and RFS between SLNB group and ALND group, and the side effects of radiotherapy after SLNB were less than those of ALND group (13). Joo et al. compared the ALND group with the SLNB group in breast cancer patients with 1-3 metastatic SLNs after mastectomy (20). The study found that ALND did not improve the survival outcome of patients with pN1 breast cancer after mastectomy. Some scholars have found that without high risk factors, ALND or radiotherapy instead of ALND does not worsen DFS (28). This shows the surgical method shift from ALND to SLNB and even omitting axillary surgery in selected patients is reasonable. There is currently insufficient evidence in SLN-positive patients after mastectomy, and supplemental axillary radiotherapy may be considered if ALND is not performed. It is expected that more high-level studies will contribute to the refinement of axillary management in early breast cancer patients. In patients clinically diagnosed with positive axillary lymph nodes who have not received neoadjuvant therapy, SLN micrometastasis after breast-conserving surgery can safely exempt from ALND and axillary radiotherapy. After breast-conserving surgery, patients with 1-2 macrometastases of SLN can receive whole breast or high tangential field irradiation without ALND and patients with more than 2 metastases of SLN or high risk of non-SLN metastasis need additional axillary radiotherapy after ALND exemption. However, only about one third of the patients had non-SLN metastases which could truly benefit from ALND (23). As the non-SLN status can only be determined by ALND, it has become crucial to predict the non-SLN status in patients with positive SLN. More recently, axillary radiotherapy has become an alternative to ALND for patients with low burden positive SLNB. Our research can be used to predict non-SLN metastasis in T1-2 and HR+ breast cancer with 1-3 positive SLNs. The availability of nomogram would greatly aid the clinicians in predicting risk of non-SLN metastasis and identify patients at high risk of non-SLN metastasis that might benefit from ALND, and those low risk patients could avoid overtreatment and procedural complications.

Previous studies have shown that tumor multifocality in breast-conserving patients was significantly associated with non-SLN metastases (29, 30). One possible explanation is that lymph containing tumor cells drains from multiple sites into the ipsilateral axilla. In our study, tumor multifocality was not associated with non-SLN metastases. The result might be related to different types of breast surgery. At present, there was still controversy regarding non-SLN metastases and ER expression. In HR+ HER2− breast cancer, multiple studies have shown relatively high risk of late relapse, with more than 50% of recurrences occurring after 5 years. Triple negative and HER-2 positive breast cancer are more aggressive, and demonstrates a high rate of recurrence at an earlier time point following initial treatment. As is well known, HR-positive breast cancers carry a better prognosis for disease-free survival and overall survival than triple negative and HER2-positive breast cancer patients. Breast cancer is a heterogeneous disease, with diverse subtypes, each driven by distinct molecular and genetic mechanisms, which may lead to different clinical decision and management. So we’re looking for this segment of the patient population to avoid axillary lymph node dissection. In our study, ER expression was negatively related with non-SLN metastases. Yu et al. found that ER status are not associated with the risk of metastasis (8). Some other studies have found that ER-positive breast cancers tend to have non-SLN metastasis more frequently (31, 32). For further verification, multicenter randomized controlled studies are needed. We found that younger patients had a higher likelihood of developing non-SLN metastasis. It is consistent with some previous research (33). To determine the grade of breast cancer, the Nottingham grading system (NGS) has been widely used by various professional bodies internationally. The grade of an individual tumor is observed from an assessment of three morphological features, each of which is scored 1-3 (34). In our study, HS is the indispensable parameter in predicting tumor aggressiveness and prognosis of patients.

With the development of SLNB, incidence rate of postoperative complications of breast cancer has been significantly reduced. Even though postoperative lymphedema affects a small proportion of patients, its development greatly affects their quality of life (6, 23). In view of this, preoperative imaging for lymph node evaluation is highly recommended. In China, ultrasound is an important tool to assess axillary lymph nodes preoperatively. A recent systematic review analyzed 17 studies of axillary ultrasound, showed the sensitivity and specificity of ultrasound was 26%-76% and 88%-98% according to morphological criteria (12, 35–37). In this study, preoperative ultrasound was used to detect lymph node load with a satisfactory accuracy. We found that cortical thickness of lymph node was an independent risk factor for non-SLN metastatic involvement. This result is in accordance with the results of PU HAN et al (38).

Over the past century, breast cancer survival rates have continuously improved due to advances in adjuvant treatments. The clinicopathological factors affecting patients’ LRR and DM have changed. Preoperatively predicting the risk of non-SLN metastasis in patients with 1-3 positive SLNs profoundly shapes clinical medical decision making. Nevertheless, our research still has shortcomings. This is a single-center retrospective research that may has the selection bias. More significantly, it’s a pity that we failed to design randomized controlled trials to understand the prognosis of SLNB+ patients stratified by postmastectomy radiotherapy (PMRT)or ALND. Additional external validation cohorts are urgently demanded to further evaluate the feasibility of our research.

## Conclusion

Our study present that younger age, higher HS, cortex thickening of lymph nodes, and lower levels of ER expression were significantly associated with non-SLN metastasis in T1–2 and HR+ breast cancer patients with 1-3 positive SLNs after mastectomy. The availability of the nomogram would greatly aid the clinicians in predicting risk of non-SLN metastasis and identify patients at low risk which ALND may be omitted.

## Data availability statement

The original contributions presented in the study are included in the article/supplementary material. Further inquiries can be directed to the corresponding authors.

## Ethics Statement

Ethical approval was waived by the local Ethics Committee of the Chongqing Medical University in view of the retrospective nature of the study and all the procedures being performed were part of the routine care. Written informed consent for publication was obtained from all participants.

## Author contributions

KX, YF, GY organized the database. JC, YM, HC, JY, and YH performed the statistical analysis. All authors contributed to the conception and design of the study and wrote the first draft and sections of the manuscript. All authors contributed to the article and approved the submitted version.
